# Insights into the complete genomes of carbapenem-resistant *Acinetobacter baumannii* harbouring *bla*_OXA-23,_ *bla*_OXA-420_ and *bla*_NDM-1_ genes using a hybrid-assembly approach

**DOI:** 10.1099/acmi.0.000140

**Published:** 2020-06-05

**Authors:** Saranya Vijayakumar, Chand Wattal, Oberoi J.K., Sanjay Bhattacharya, Karthick Vasudevan, Shalini Anandan, Kamini Walia, Balaji Veeraraghavan

**Affiliations:** ^1^​ Christian Medical College, Vellore, India; ^2^​ Sir Ganga Ram Hospital, New Delhi, India; ^3^​ Tata Medical Center, Kolkata, India; ^4^​ Indian Council of Medical Research, New Delhi, India

**Keywords:** *Acinetobacter baumannii*, bla_OXA-420_, *bla*_CARB-2_, IS*Aba3*, Int1

## Abstract

Carbapenem resistance in *Acinetobacter baumannii* is due to *bla*_OXA-23_, which is endemic in India. Recently, the sporadic presence of *bla*_OXA-58_ as well as the occurrence of dual carbapenemases were observed. The mobility as well as the dissemination of these resistance genes were mainly mediated by various mobile genetic elements. The present study was aimed at characterizing the genetic arrangement of *bla*_OXA-23,_ *bla*_NDM-1_ and *bla*_OXA-58_ identified in two complete genomes of carbapenem-resistant *A. baumannii* (CRAB). Complete genomes obtained using a hybrid-assembly approach revealed the accurate arrangement of Tn*2006* with *bla*_OXA-23,_ IS*Aba125* with *bla*_NDM_ and IS*Aba3* with *bla*_OXA-58._ In addition, the association of *IntI*1 integrase with the *bla*_CARB-2_ gene and several virulence factors required for type-IV pili assembly, motility and biofilm formation have been identified. The current study provided deeper insight into the complete characterization of insertion sequences and transposons associated with the carbapenem-resistant genes using short reads of IonTorrent PGM and long reads of MinIon in *A. baumannii *.

## Introduction

*Acinetobacter baumannii* a nosocomial pathogen is of particular concern due to the global occurrence of multi-drug-resistant (MDR) and pan-drug-resistant (PDR) strains [1]. *A. baumannii* is a leading cause of various health-care-associated infections like blood-stream infections, ventilator-associated pneumonia, urinary tract infections and wound infections [2]. *A. baumannii* has acquired resistance to an array of antimicrobial agents belonging to different classes of antibiotics like cephalosporins, carbapenems, aminoglycosides, fluoroquinolones, chloramphenicol and tetracyclines [3]. Carbapenems belong to the β-lactam class of antimicrobials [4]. According to Ambler’s system of classification, β-lactamases are classified into four classes: A, B, C and D [4]. Further, classes A, C and D are termed as serine β-lactamases whereas class B is called metallo-β-lactamases [4].

Carbapenems are the last resort drugs to treat *A. baumannii* [4]. Therefore, the development of resistance to carbapenem is highly concerning [4]. Carbapenem resistance in *A. baumannii* is most predominantly mediated by oxacillinases (OXAs) like *bla*_OXA-23_ like, *bla*_OXA-24_ like, *bla*_OXA-58_ like and metallo-β-lactamases (MBLs) like *bla*_NDM_ like, *bla*_IMP_ like, *bla*_VIM_ like and *bla*_SIM_ like [5]. The sporadic presence of other carbapenemases like *bla*_KPC_ like and *bla*_GES_ like have also been reported [6, 7]. A recent study by Nordmann *et al*. reported region-wise carbapenem non-susceptibility rates in *A. baumannii,* which have ranges as follows : (i) Asia-Pacific – 55–91 %; Europe – 58–85 %; North America – 32–50%; and Latin America – 53–90 % [8]. In India, results from recent studies reported 40–75% carbapenem resistance in *A. baumannii* [9]. *bla*_OXA-23_ like is the most commonly reported carbapenemase followed by *bla*_NDM_ like in *A. baumannii* strains [10]. In *A. baumannii* carbapenem-resistance genes can be disseminated by mobile genetic elements (MGEs) like insertion sequences, integrons and transposons [11].

Next-generation sequencing (NGS) has facilitated the complete characterization of whole-genome sequences from microbial species [12]. In addition, whole-genome sequencing (WGS) helps in studying the dynamics and genomic evolution of bacterial pathogens [13]. In this study, hybrid assembly was performed to obtain complete genomes using long reads from MinION and short reads from IonTorrent. With this, the main objective of this current study is (i) to identify genes encoding carbapenem resistance and (ii) to decipher the genetic arrangement of insertion sequences and integrons, which are involved in the mobilization and dissemination of resistance genes among CRAB.

## Methods

### Bacterial isolates and identification

Two clinical isolates, ACN21 from Sir Ganga Ram hospital, Delhi NCR and CIAT758 from Tata Medical Center, Kolkata collected as a part of Indian Council of Medical Research (ICMR) surveillance study in the year 2018 were included in this study. Both isolates were identified as *A. baumannii-calcoaceticu*s complex (*Acb* complex) using standard biochemical methods. *bla*_OXA-51_ PCR was performed to confirm both isolates as *A. baumannii* [14].

### Anti-microbial susceptibility testing (AST)

ACN21 and CIAT758 were subjected to AST to determine the MIC of imipenem and meropenem using broth micro-dilution (BMD) and interpreted as per The Clinical and Laboratory Standards Institute (CLSI) guidelines [15].

### Multiplex PCR for anti-microbial resistance (AMR) genes

To determine the presence of AMR genes, multiplex PCR for class A, B and D carbapenemases was performed [5]. Known positive control for appropriate genes were included.

### Next-generation sequencing

#### Short-read sequencing using IonTorrent PGM

DNA was isolated from pure cultures using Qiagen DNA Mini Kit (QIAGEN, Hilden, Germany). Whole-genome shotgun sequencing was performed using Ion Torrent PGM TM platform with 400 bp chemistry (Life Technologies, Carlsbad, CA, USA) as per the manufacturer’s instructions.

#### Long-read sequencing using MinIon

Long-read sequencing was performed using MinIon Oxford Nanopore sequencing. DNA library preparation was constructed using SQK- LSK108 Kit R9 version (Oxford Nanopore Technologies, Oxford, UK) using the 1D sequencing method for sequencing of genomes according to the manufacturer’s protocol (https://nanoporetech.com/resource-centre/protocols). FLO-MIN106 R9 flow cell in a MinION Mk 1B sequencer was utilized for sequencing. The sequencing was conducted upto 48 h and raw fast5 files were generated using MinKNOW software ver. 1.15.1.

### *De novo* assembly of genomes

#### Short-read assembly

AssemblerSPAdes version 5.0.0.0 embedded in Torrent Suite Server v.5.0.3 was employed for short-read error correction and assembly. Subsequently, the quality metrics of resulting fragmented genome with multiple contigs were analysed.

### Long-read assembly

#### Reads processing and base calling

The raw fast5 files generated from sequencing were subjected to basecalling using ONT Albacore Sequencing Pipeline Software version 2.0.2 (https://nanoporetech.com/about-us/news/new-basecaller-now-performs-raw-basecalling-improved-sequencing-accuracy). Further, the genomes were separated according to the barcodes and resulting fastq files were merged for subsequent analysis.

#### Canu assembly and polishing

Low-quality reads often lead to mis-assemblies and frame-shifted INDELS. The low-quality reads were removed using Nanofilt v2.5 (https://github.com/wdecoster/nanofilt). The filtered fastq files are then error corrected and trimmed with canu v1.7 [16] to further increase the quality of reads. Further, the trimmed reads were assembled using canu assembly option incorporated in canu v1.7. The long-read assembly generally contains a significant amount of errors and INDELS. Hence, long-read assemblies were polished using Nanopolish version 0.10.1 (https://github.com/jts/nanopolish) to reduce the inconsistency of the assembly. Initially, the genomes were split into fragments 50 kb each and individually polished in parallel. Then the polished fragments were merged to obtain complete genome assembly.

#### Hybrid assembly and quality assessment

Hybrid assembly incorporating both IonTorrent and MinION reads was performed using Unicycler (v0.4.6) [17]. Initially, the short reads were error corrected with different k-mers using SPAdes [18] to filter out low-quality reads. In addition, long-read assembly and polishing was performed using Miniasm v0.3 [19] and Racon v1.3.3 [20], respectively. Further, both long-read and short-read assemblies were bridged to generate genome assembly. Finally, repeated short-read polishing steps with Pilon [21] were performed to generate a complete and accurate genome assembly. The assembly statistics and other quality metrics were analysed using Quast [22] to verify the quality, completeness and contiguity of the genomes.

#### Genome annotation

Genome annotation was performed using NCBI Prokaryotic Genome Annotation Pipeline (PGAP) (www.ncbi.nlm.nih.gov/genome/annotation_prok/) and the PATRIC database (http://www.patricbrc.or g) [23]. Antimicrobial resistance genes were identified using ResFinder v3.0 (https://cge.cbs.dtu.dk//services/ResFinder/) [24]. Virulence factors were detected using the Virulence Factors Database, VFDB [25]. Insertion sequences and the presence of prophage-related sequences were screened with IS Finder (https://www-is.biotoul.fr/blast.php) and the PHAST tool, respectively [26, 27]. Genomic Island was detected using Island Viewer 4 with the SIGI-HMM algorithm [28]. Sequence types of the isolates were identified with MLST 2.0 tool (multi-locus sequence typing) [29].

## Results and discussion

Both isolates were confirmed as *A. baumannii* by the presence of intrinsic, *bla*_OXA-51_-like gene. Both phenotypically and genotypically, ACN21 and CIAT758 were confirmed as carbapenem resistant. However, they differ only in the type of carbapenem resistance gene they harbour. The MIC of imipenem and meropenem were determined as 128 µg ml^−1^ and 256 µg ml^−1^ for ACN21, respectively, whereas for CIAT758 the MICs for both were 128 µg ml^−1^. Multiplex PCR revealed the presence of *bla*_NDM_ like and *bla*_OXA-58_ like in ACN21 and *bla*_OXA-23_ like and *bla*_OXA-58_ like in CIAT758.

Hybrid assembly of ACN21 revealed the presence of one chromosome of size 3 827 138 bp with eight plasmids ranging from 116 047–5734 bp size whereas CIAT758 had a single chromosome with 4 017 696 bp size and three plasmids of 78 125, 47 417 and 29 128 bp size. The genome features of the isolates were mentioned in Table 1.

**Table 1.** Genomic features and resistance genes identified in *A. baumannii* strains ACN21 and CIAT758

Strain name	ACN21		CIAT758	
Genomic size	3 827 138 bp		4 017 696 bp	
Total coding sequences	3822		4040	
Total pseudo genes	183		277	
GC content	38.88		39.02	
**Anti-microbial resistance genes**	**Gene**	**Location**	**Gene**	**Location**
	NDM-1*	Chromosome	*OXA-68*	Chromosome
	*ADC-25*	Chromosome	OXA-23*	Chromosome
	*OXA-94*	Chromosome	*ADC-25*	Chromosome
	*mph(E*)	Chromosome	OXA-58*	Plasmid 1
	*msr(E*)	Chromosome	*msr(E*)	Plasmid 1
	*Sul1*	Plasmid 2	*mph(E*)	Plasmid 1
	*armA*	Plasmid 2	*tet(39*)	Plasmid 1
	CARB-2*	Plasmid 2	*aac(3)-IId*	Plasmid 1
	*OXA-420*	Plasmid 2	*Sul1*	Plasmid 2
	*mph(E*)	Plasmid 2	*aph(3’)-VIa*	Plasmid 2
	*msr(E*)	Plasmid 2	*PER-7*	Plasmid 2

**Fig. 1.**
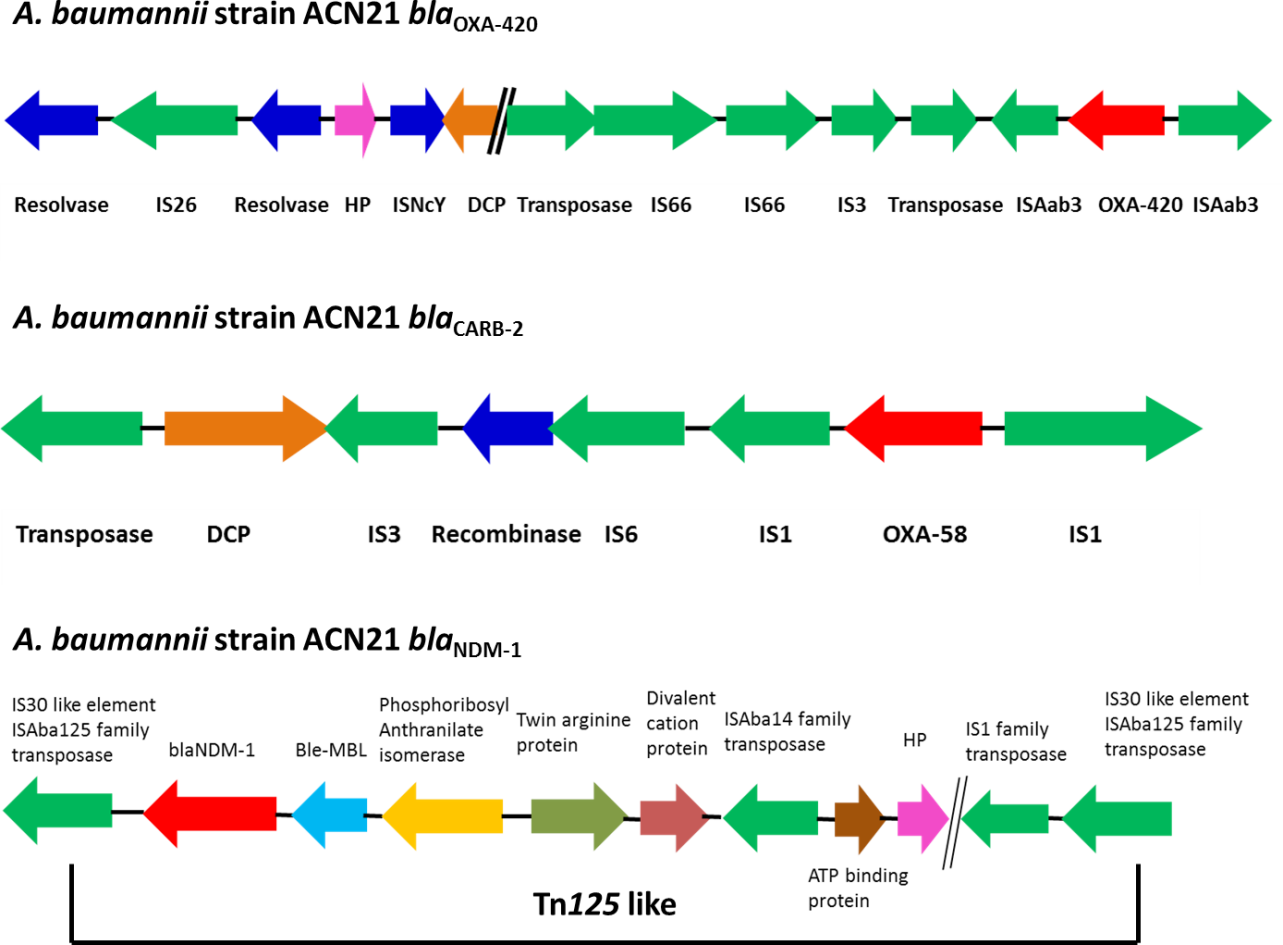
Graphical representation of genetic arrangement of *bla*_OXA-420_, *bla*_CARB-2_ and *bla*_NDM-1_ in *A. baumannii* strain ACN21. The direction of arrows indicates the orientation of ORFs.

**Fig. 2.**
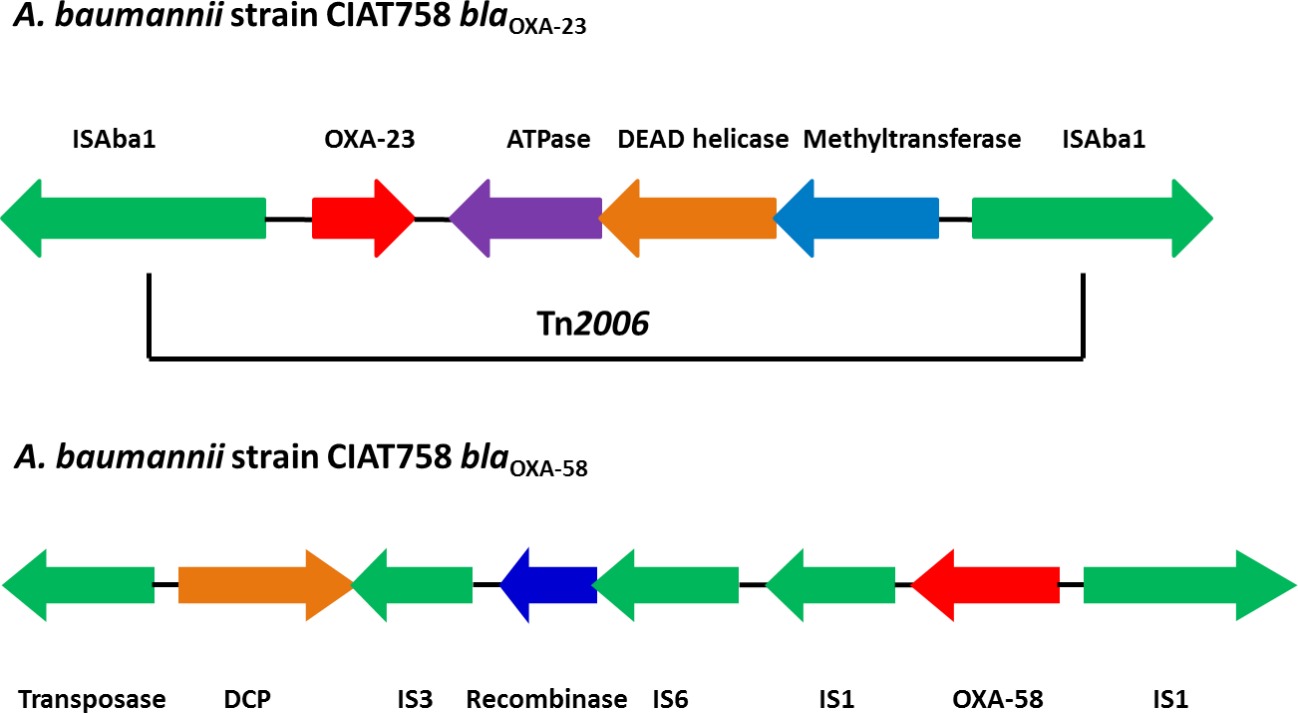
Graphical representation of genetic arrangement of *bla*_OXA-23_ and *bla*_OXA-58_ in *A. baumannii* strain CIAT758. The direction of arrows indicates the orientation of ORFs.

*Genetic arrangement of these resistance genes were shown in Figs 1 and 2.

Ion Torrent short-read assembly produced accurate genome with multiple fragments. In contrast, MinIon Nanopore long-read assembly generated a single chromosomal contig but with higher frame-shifted INDELS (>30 %) even after repeated long-read polishing that makes downstream analysis difficult. Further, the repeated polishing with short reads generated a highly accurate single chromosomal contig with reduced frame-shifted INDELS (<10 %), which enabled accurate analysis of the AMR and other MGEs.

Long-read-sequencing technologies such as Pacific Biosciences (PacBio) and Oxford Nanopore will be helpful in generating longer reads, which in turn will allow the gaps to be covered [30]. MinIon only reads may be inclined to errors, however that can be efficiently overcome by combining with short-read sequencers like IonTorrent and Illumina [30].

The genome of ACN21 has been deposited in GenBank with accession number CP038644 for chromosome and CP038645–CP038652 for plasmids 1–8. For CIAT758, the chromosome and three plasmids were deposited with accession numbers CP038500 and CP038501, CP038502, CP038503, respectively.

### Resistance determinants

Multiple resistant genes conferring resistance to various antimicrobial classes were identified in both ACN21 and CIAT758 genomes. The genes that may contribute resistance to aminoglycosides, beta lactams, macrolides, lincosamide streptogramin B, sulphonamide and tetracycline are listed in Table 1.

In *A. baumannii *, carbapenem resistance is predominantly due to acquired class-D *bla*_OXA-23_ followed by other oxacillinases and metallo-beta lactamases [1]. The genome ACN21 was devoid of *bla*_OXA-23_, while the presence of *bla*_NDM-1_ and *bla*_OXA-420_ could be encoding for carbapenem resistance. In ACN21, *bla*_NDM-1_ resides in chromosome and *bla*_OXA-420_ in plasmids whereas previous studies reported the co-presence of *bla*_NDM_ and *bla*_OXA-58_ on the same plasmid [31–34]. In CIAT 758, *bla*_OXA-58_ was carried on the plasmid (Table 1). Presence of *bla*_OXA-23_ and *bla*_OXA-58_ in six CRAB isolates was previously reported by Principe *et al*. [35]. El Bannah *et al*. reported that 86 % of carbapenem resistance could be due to the presence of either *bla*_OXA-23_ alone or *bla*_OXA-23_ together with *bla*_KPC_ [36]. However, in this study *bla*_OXA-23,_ *bla*_OXA-58_ and *bla*_NDM-1_ was present, but *bla*_KPC_ was not observed.

### Mobile genetic elements

The main characteristic feature of *A. baumannii* is its ability to acquire, retain and disseminate multiple resistance mechanisms by combining resistance genes with an array of MGEs like insertion sequences, resistance islands and bacteriophages, which mediate the exchange of genetic material, rearrange bacterial genomes and provides an endless source of genetic adaptability [11]. Increased resistance to carbapenems due to upstream insertion of *bla*_OXA-51_ and *bla*_OXA-23_ with IS*Aba1*, *bla*_OXA-58_ with IS*Aba3* and *bla*_NDM_ with IS*Aba125* were reported from previous studies [37–39]. In this study, IS Finder analysis revealed the presence of various insertion sequences belonging to IS family IS1, IS3, IS4, IS5 and IS30 in both ACN21 and CIAT758 genomes. Phage analysis identified three phage regions (one intact, one incomplete and one questionable) in the ACN21 genome and one intact phage region in CIAT758 genome. Further analysis using Island viewer revealed the presence of six and nine genomic islands in ACN21 and CIAT758 genomes, respectively.

### Genetic context of resistance genes

#### ACN21

In the ACN21 genome, the variant of the *bla*_OXA-58_ gene, *bla*_OXA-420_ is present. The upstream of the plasmid-carried *bla*_OXA-420_ gene has truncated IS*Aba3* belonging to the family IS1. Immediately downstream from the *bla*_OXA-420_ gene, another truncated IS*Aba3* element was present. The *bla*_OXA-420_ gene is bracketed by two IS*Aba3* elements thereby forming a composite transposon (Fig. 1). Earlier studies report on the association of *bla*_OXA-58_ with IS*Aba3* [40, 41].

An acquired extended spectrum beta lactamase unusual in *A. baumannii *, *bla*_CARB-2_ (carbenicillinase gene) has been identified in this genome, which has been initially reported in GenBank from China but unpublished. The *bla*_CARB-2_ gene was first reported in a plasmid from *P. aeruginosa* in 1991 by Huovinen and Jacoby [42] and known as *Pseudomonas *-specific enzyme-1 (*PSE*-1). Variants of the *bla*_CARB_ gene, *bla*_CARB_ - 4, 5, 8 and 10 have been reported earlier in *A. baumannii *. Kamolvit *et al*. reported the presence of the *bla*_CARB-2_ gene in *A. pittii* with upstream presence of truncated class 1 integrase gene (*intI*1) [43]. In the ACN21 genome, though the *bla*_CARB-2_ gene has upstream presence of *intI*1, it is not truncated (Fig. 1).

The ACN21 genome has the *bla*_NDM-1_ gene with downstream IS*Aba125*, upstream ble-MBL gene and bracketed by two copies of IS*Aba125*. However, an additional insertion sequence, IS*Aba14* was identified upstream ATP binding protein thereby forming a Tn*125*-like composite transposon, which could be a novel finding. This is similar to the report documented by Poirel *et al*. and Jones *et al*. [44, 45]. The genetic arrangement of *bla*_NDM-1_ gene is shown in Fig. 1.

#### CIAT758

The CIAT758 genome harbours *bla*_OXA-23_ on the chromosome and bracketed by two copies of the IS4 family insertion element, IS*Aba1* (Fig. 2). A similar scenario was documented by several other studies [1, 5, 46].

In addition, CIAT758 harbours the *bla*_OXA-58_ gene with two copies of IS*Aba3* with one copy present downstream being truncated. Downstream of IS*Aba3* possess another insertion element, IS*1008* belonging to IS6 family transposase (Fig. 2). Chen *et al*. reported the presence of *bla*_OXA-58_ with truncated IS*Aba3* and IS*1008,* which provides two independent promoters for the transcription of *bla*_OXA-58_ gene [47]. Further, the deletion of promoters provided by IS*1008* results in decreased transcription of the *bla*_OXA-58_ gene and was not noticed in this genome [47].

#### Virulence factors

Several virulence factors required for pathogenesis have been identified in *A. baumannii* [12]. Both the sequenced genomes harbour genes required for assembly of type-IV pili (pilB and pilF), for twitching motility (pilT and pilU), surface polysaccharides (pgaA/B/C/D), which is involved in the synthesis of poly β-(1-6)-*N*-acetyl glucosamine for biofilm formation and protects the bacteria against innate host defenses [48, 49], outer membrane protein (OmpA) involved in pathogenesis and signal processing [50], phospholipase C, an important factor in cellular damage [51] and phospholipase D, important for human serum resistance and epithelial cell invasion, siderophores (bauA and basD), encodes for acinetobactin transport and biosynthesis, respectively [11]. Additionally, ACN21 possess the csuE gene, which belongs to the CsuA/BABCDE chaperone usher complex responsible for pili production and biofilm formation [52] whereas CIAT758 possesses the biofilm-associated protein (Bap) required for the development of mature biofilm structures [53].

#### Multi-locus sequence typing (MLST)

Currently, in *A. baumannii *, epidemiological characterization of clinical isolates has been described with eight international clonal lineages (IC-1 to IC-8). Among these, IC-1, IC-2 and IC-3 were the most common clonal lineages reported from various countries [54]. The majority of outbreaks due to CRAB were reported to be associated with isolates belonging to the IC-2 lineage [10].

*In silico* analysis of MLST using Oxford scheme identified ACN21 with ST1089. Uwingabiye *et al*. reported the presence of ST1089 and also mentioned that this ST was first reported in India in 2015 from PGIMER, Chandigarh [55]. Unlike other STs, ST195, which was widely reported from Asian countries and European Nations, ST1089 is very rare. While in CIAT758, ST585 was identified and belongs to International Clone 8 (IC-8). ST585 is the single loci variant (SLV) of ST391, which was first reported in India by Rynga *et al*. [56]. Another study from Egypt showed that the study isolates belonging to ST391 carries *bla*_OXA-23_ alone or *bla*_NDM_ alone or *bla*_OXA-23_ with *bla*_KPC_, which is in contrast to the current study where CIAT758 belongs to ST589 and carries *bla*_OXA-23_ with *bla*_OXA-58_ [36]. Such findings indicate that despite diverse sequence types, *bla*_OXA-23_ is the major contributor of carbapenem resistance in *A. baumannii* worldwide. As discussed by El Bannah *et al*., certain STs are endemic in particular countries whereas others belong to a worldwide clonal complex and disseminate globally [36]. Similarly, a previous study by the same authors reported ST208 as the endemic sequence type, which is a SLV of globally disseminated ST92 [57].

### Novel findings using hybrid assembly

In *A. baumannii *, MGEs like insertion sequences and integrons play a major role in the dissemination of anti-microbial resistance genes. Short reads from Ion Torrent sequencing results in a greater number of contigs, which leads to difficulty in deciphering the appropriate arrangement of MGEs. Hybrid assembly provides the complete genome, which is helpful in identifying the association of insertion sequences/integrons with resistance genes. In ACN21, the association of *IntI*1 integrase with the *bla*_CARB-2_ gene was observed in the complete genome using a hybrid-assembly approach, whereas this association was completely missing in short-read genome assembly. Similarly, in the CIAT758 genome the presence of insertion element, IS*Aba1,* was observed in duplicates, whereas the hybrid genome assembly approach enabled the complete and accurate structural arrangement to be revealed.

### Limitations of the study

Only two complete genomes of CRAB were characterized in this study. Characterization of the clinical isolates of more complete genomes of *A. baumannii* with different variants of the above mentioned resistance genes will be helpful in the comprehensive understanding of the variations in their genetic arrangement.

### Future recommendations

Whole-genome sequencing of *A. baumannii* using the hybrid-assembly approach in the upcoming studies are necessary. Such studies will be helpful to gain thorough knowledge regarding various antibiotic resistance genes and the role of respective MGEs that are involved in the dissemination of the same.

## Conclusion

In this study, complete genomes of two CRAB, ACN21 and CIAT758 were characterized using the hybrid-assembly approach. This study deciphers the genetic arrangement of *bla*_OXA-23_ with Tn*2006*, *bla*_NDM-1_ with a Tn*125*-like transposon, *bla*_OXA-420_ with IS*Aba3* and *bla*_CARB-2_ with class 1 integron. A novel Tn*125*-like transposon carrying *bla*_NDM-1_ was identified in the complete genome of ACN21. One limitation with this study is that only two complete genomes were characterized. However, such findings using complete genomes will be helpful in studying the genetic backbone of significant resistance genes and also in exploration of associated novel MGEs.
